# Infectious pancreatic necrosis virus (IPNV) from salmonid fish enters, but does not replicate in, mammalian cells

**DOI:** 10.1186/1743-422X-9-228

**Published:** 2012-10-05

**Authors:** Irene Ørpetveit, Thomas Küntziger, Hilde Sindre, Espen Rimstad, Birgit H Dannevig

**Affiliations:** 1 Norwegian Veterinary Institute, P.O. Box 0750, Sentrum, Oslo, NO, 0106, Norway; 2 Institute of Basic Medical Sciences, Faculty of Medicine, University of Oslo, Box 1110, Blindern, Oslo, NO, 0317, Norway; 3 Department of Food Safety and Infection Biology, Norwegian School of Veterinary Science, Box 8146, Dep, Oslo, NO, 0033, Norway

**Keywords:** Host susceptibility, Virus entry, Entry mechanism, Receptor, Birnaviridae, Aquabirnavirus, IPNV

## Abstract

**Background:**

The aquatic birnavirus infectious pancreatic necrosis virus (IPNV) causes infectious pancreatic necrosis (IPN), a severe disease in farmed salmonid fish. IPNV has a very broad host range and infects many different species of fish as well as molluscs and crustaceans. Investigation of the host reservoir of a virus may reveal important molecular mechanisms governing the infection processes such as receptors and entry mechanisms. In the present work we have studied whether IPNV is able to infect cells with different mammalian origin.

**Results:**

IPNV bound in a specific manner to a membrane protein of the rabbit kidney cell line RK-13 as shown by the use of a virus overlay protein binding assay (VOPBA). Six different mammalian cell lines were inoculated with IPNV and incubated in parallels at different temperatures. At 7 days post inoculation (dpi), IPNV was detected by indirect immunofluorescent antibody test (IFAT) in all the cell lines. Confocal microscopy confirmed intracellular presence of the virus. No apparent cytopathic effect (cpe) was observed in any of the cultures, and no viral replication was demonstrated with real-time RT-PCR.

**Conclusion:**

Our results show that IPNV is able to enter into a wide range of mammalian cells, and virus entry is most likely receptor mediated. We found no indication of IPNV replication in any of the mammalian cell lines tested.

## Introduction

Infectious pancreatic necrosis virus (IPNV) is the etiological agent of infectious pancreatic necrosis (IPN), an important disease in the salmonid fish farming industry. The virus is icosahedral, naked, with a bisegmented dsRNA genome and belongs to genus *Aquabirnavirus* in the family *Birnaviridae*. The genus *Aquabirnavirus*, of which IPNV is the type species, comprises aquatic birnaviruses isolated from a large number of fish species living at different temperatures from 4°C to 28°C [1]. In addition to the genus *Aquabirnavirus*, the family *Birnaviridae* also includes the genus *Avibirnavirus*, type strain *Infectious bursal disease virus* (IBDV) infecting poultry, the genus *Entomobirnavirus*, type strain *Drosophila X virus* (DXV) infecting the fruit fly (*Drosophila melanogaster*) [1] and the recently added genus *Blosnavirus*, type strain *Blotched snakehead virus* (BSNV) [2].

The host range of aquatic birnaviruses is not restricted to fish, as viruses from this genus have also been isolated from shrimps [3], crustaceans and molluscs [4]. IPNV replicates in cell lines derived from a wide range of freshwater and marine hosts [5], and even in cell lines derived from green turtles [6]. While IPNV propagation in cell culture is shown to have an upper temperature limit of 28°C [7], the RNA dependent RNA polymerase (RdRp) is active at 37°C as assessed by *in vitro* RNA synthesis experiments [8]. The virus is temperature resistant, and retains infectivity even after one hour at 60°C in fish silage [9].

To our knowledge, IPNV is not reported to replicate in cell lines derived from homeothermic animals or insects. This may be attributed to properties of either the host or the virus or of both. The fish virus betanodavirus Red grouper nervous necrosis virus (RGNNV) was shown to bind to human cells in cell culture, but was unable to enter into these cells. However, virus replication could be initiated by transfection of cells with viral RNA [10].

Knowledge of possible virus host reservoirs is important for understanding the infection mechanisms of the viruses, such as viral receptors, entry mechanisms and temperature limits for viral replication. We have investigated whether IPNV can adsorb to, enter into and replicate in mammalian cell lines.

## Materials and methods

### Cells and virus

The piscine and mammalian cell lines applied are listed in Table 1. Chinook salmon embryo (CHSE)-214 cells are piscine cells susceptible to IPNV, and CHSE-214 is one of the preferred cell lines for isolation and propagation of the virus. The mammalian cell lines used were rabbit kidney (RK)-13 cells, foetal bovine turbinate (FBT) cells, monkey kidney epithelial Vero cells, primary human embryonic fibroblasts (HE cells), human lung carcinoma cells (A549) and human leukemic monocytes (THP-1 cells).

**Table 1:** Cell lines

**Cell lines**	**Growth media**^(^*^)^
Chinook salmon embryo cells (CHSE)-214 (Fryer et al., 1965)	Ørpetveit et al., 2008
Rabbit kidney (RK)-13 cells (ATCC CCL-37)	MEM Eagle’s with Earle’s BSS (EMEM) supplemented with 10% FBS, 4 mM L-glutamine and 50 μg/ml gentamicin, final pH 7.6
Foetal bovine turbinate (FBT) cells (“in house” cell line)	As described for RK-13 cells, but supplemented with 16.4 mM Tris-buffer (pH 7,8) and 5.3 mM NaHCO_3_, final pH 7.6
Vero cells ATCC CCL-81^TM^	RPMI-1640 (Sigma-Aldrich, St Louis, MO) supplemented with 10% FBS and 50 μg/ml gentamicin.
Primary human embryonic fibroblast (HE) cells (a kind gift from Rikshospitalet University Hospital, Oslo, Norway)	
A549 cells (human) (ATCC CCL-185^TM^)	
THP-1 cells (human) (ATCC TIB-202^TM^)	

^(^*^)^ Where not otherwise indicated, the cell culture reagents were obtained from Cambrex Bio Science, Rockland, ME.

Two IPNV preparations were applied: a first passage virus supernatant (IPNV-1p) and purified IPNV (IPNV-pur). Kidney tissue from an experimentally IPNV infected Atlantic salmon (*Salmo salar L.*) (IPNV serotype Sp, a kind gift from VESO-Vikan, Norway) was homogenized in T10 (Table 1) and cleared by low speed centrifugation. This tissue supernatant was passaged once in BF-2 cells, and the resulting cell supernatant is referred to as IPNV-1p. IPNV-pur was prepared from IPNV-1p by CsCl density-gradient centrifugation as previously described [11]. The virus preparations were aliquoted and stored at −80°C until use.

Virus titration was performed by end-point dilution as described [11], and TCID50 ml^-1^ was determined according to Kärber (1931) [12].

Prior to virus inoculation, all cells were grown to 70–80% confluence. Cell culture medium without FBS was used as virus dilution medium to obtain the desired titres. After virus adsorption for 1 h at 37°C, the cells were incubated at 15°C, 25°C or at 37°C until harvesting/fixation.

### Virus overlay protein binding assay (VOPBA)

Membrane proteins were extracted from cells as previously described [11]. Binding of IPNV to membrane proteins from RK-13 (Table 1), was studied by VOPBA as previously described [11]. A fraction of CHSE-214 (Table 1) membrane proteins was included as positive control. In brief, approximately 20 μg of membrane proteins extracted from the respective cell lines were separated by SDS-PAGE and transferred to a 0.45-μm nitrocellulose membrane (blot) using the Criterion Precast Gel System (Bio-Rad). After blocking, the blot was incubated for 2 h at room temperature with IPNV-pur (final titre >10^6^ TCID_50_ ml^-1^). Bound virus was detected with a monoclonal antibody against virus protein (VP)-3 (anti-VP3) (IPN-VP3-C12, Intervet Norbio, Bergen, Norway) and a HRP-conjugated sheep anti-mouse Ig (Amersham Biosciences, Bucks, UK) as secondary antibody. Negative control blots were prepared similarly, except that IPNV-pur was replaced with PBS. To verify the reactivity of the primary antibody against the virus protein, IPNV proteins were included in the SDS-PAGE.

### Examination of inoculated cell cultures

#### Indirect immunofluorescence test (IFAT)

Mammalian cells (Table 1) were grown in 96-well strip plates (Corning® Costar®, Corning, NY). Each well contained 150 μl of cell culture medium, into which 50 μl of IPNV-pur (10^6^ TCID_50_ ml^-1^) was added. Parallel plates with each cell culture were incubated for one week at 15°C, 25°C and 37°C, respectively. Cells were fixed with 80% acetone and probed with monoclonal primary antibodies against virus protein (VP) 2 (anti-VP2) (1:50, anti-VP2 N1-H8, Intervet Norbio, Bergen, Norway), or against virus protein (VP) 3 (anti-VP3) (1:50). As secondary antibody, we applied FITC-conjugated anti-mouse-Ig (1:100, SouthernBiotech, Birmingham, AL 35209 USA). Nuclei were stained with 10 μg/ml propidium iodine (Sigma). Fluorescence images were acquired on a Leica DM IL inverted phase contrast microscope (Leica Microsystems Wetzlar GmbH, Wetzlar, Germany) equipped with a 40X/0.5 objective and a Nikon DXM1200F digital camera.

#### Confocal microscopy

RK-13 and Vero cells were grown on glass cover slips in 24-well plates (Corning® Costar®, Corning, NY) and inoculated with IPNV-pur (10^6^ TCID_50_ ml^-1^), followed by incubation at 37°C for 24 h. Paraformaldehyde fixation and permeabilization with 0.1% Triton X-100 was performed as described [13], and cells were probed with anti-VP2 (1:50) followed by DyLight 488 secondary antibodies (1:500; Jackson lmmunoResearch West Grove, PA USA). Nuclei were stained with 0.25 mg/ml DAPI (Sigma). Confocal images were acquired on an Olympus FluoView 1000 inverted microscope equipped with a Super Apochromat 60X/1.35 oil objective.

#### Real-time reverse transcription–polymerase chain reaction (RRT-PCR)

Vero and HE cells grown in 25-cm^2^ culture flasks were inoculated with IPNV-1p (final virus titre 10^4^ TCID_50_ ml^-1^). After adsorbtion at 37°C for 1 hour, the inoculum was replaced with fresh medium, and the cells were further incubated in parallels at 25°C and 37°C. At 7, 14, 21 and 27 days post inoculation (dpi), the cells at 37°C were passaged with cocultivation (1:1) with uninfected cells. Cells incubated at 25°C were not passaged due to poor growth after 7 days. At different time points, the cells were investigated for cytopathic effect (cpe), and cell medium from both cell lines was harvested and stored at −80°C until virus titration. The cell monolayers were washed twice with 2 ml PBS, following the addition of 1.5 ml of NucliSens® easyMAG™ lysis buffer (bioMérieux, Boxtel, The Netherlands). Nucleic acid extraction was performed with the NucliSens® easyMAG™ (bioMerieux Inc.) according to the manufacturer’s instruction for the off-board protocol. Nucleic acid concentrations were determined using a NanoDrop ND-1000 (NanoDrop Technologies, Wilmington, Delaware USA), and the RRT-PCR was performed in duplicates as described [14] with 250 ng/μl of nucleic acids in each reaction.

## Results

**Figure 1:**
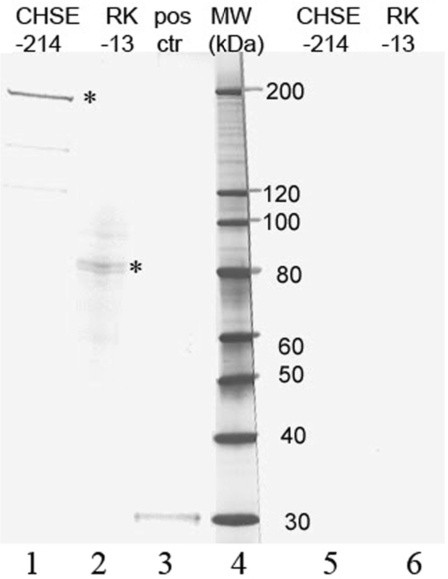
**Detection ofÂ IPNV bound to membrane proteins from CHSE-214 cells and RK-13 cells.** Membrane proteins from CHSE-214 and RK-13 cells were separated on SDS-PAGE, transferred onto a nitrocellulose membrane and incubated with IPNV-1p (TCID_50_ ml^-1^ approx. 10^5^) (lanes 1–3) or PBS without IPNV (lanes 5–6). Bound virus was detected using anti-VP3 as primary antibody. The positive control (+ctr) consisted of IPNV-1p. The asterisks indicate specific IPNV-binding proteins.

VOPBA showed that IPNV interacted in a specific manner with a membrane protein from the mammalian RK-13 cells migrating at approximately 85 kDa (Figure 1). Specific binding was also detected between IPNV and membrane proteins from CHSE-214 salmon embryo cells, with a reactive band at 220 kDa, as previously shown [11]. No bands were observed when IPNV-pur was replaced with PBS (Figure 1). A band corresponding in size with VP3 appeared in the lane with the positive control virus, thus verifying the reactivity of the primary antibody (Figure 1).

RK-13, FBT, Vero, HE, AK549 and THP-1 cells (Table 1) were inoculated with IPNV in 96-well plates and incubated in parallels at different temperatures. The cells were examined for cpe at 7 dpi and subsequently immunostained. Cells kept at 15°C and 25°C were in poor condition, but still attached to the surface, while the cells kept at 37°C had grown to confluence. Positive staining for IPNV was detected in all the investigated cell lines, regardless of incubation temperature (not shown). No IPNV positive fluorescence was seen in uninfected cells (not shown). Despite the high virus titres applied, no apparent cpe was observed in any of these cultures.

**Figure 2:**
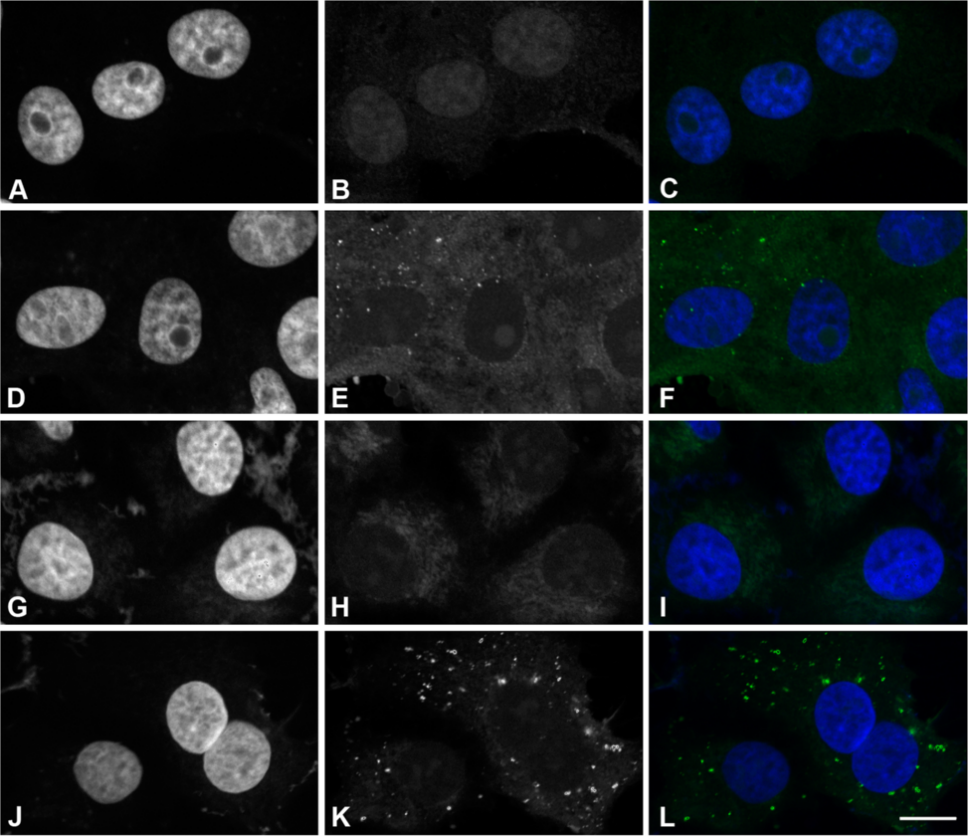
**Confocal microscopy of mammalian cell lines inoculated with IPNV.** Localization of IPNV in Vero (**A**-**F**) and RK-13 (**G**-**L**) cells that were uninfected (A-C and G-I) or IPNV infected (D-F and J-L). Cells were immunolabeled with anti-VP2 (B, E, H, K) and nuclei counterstained with DAPI (A, D, G, J). Merged pictures are shown (C, F, I, L). Bar: 10 μm.

Confocal microscope analysis confirmed the presence of IPNV in both RK-13 and Vero cells (Figure 2). The virus was detected in the cytoplasm in discrete, punctate structures over a faint cytoplasmic background, but not attached to the outside of the plasma membrane. This punctate staining, which was observed in approximately 100% of the infected RK13 cells and in 10–20% of the infected Vero cells, was not found in uninfected cells, where only cytoplasmic background could be observed.

**Figure 3:**
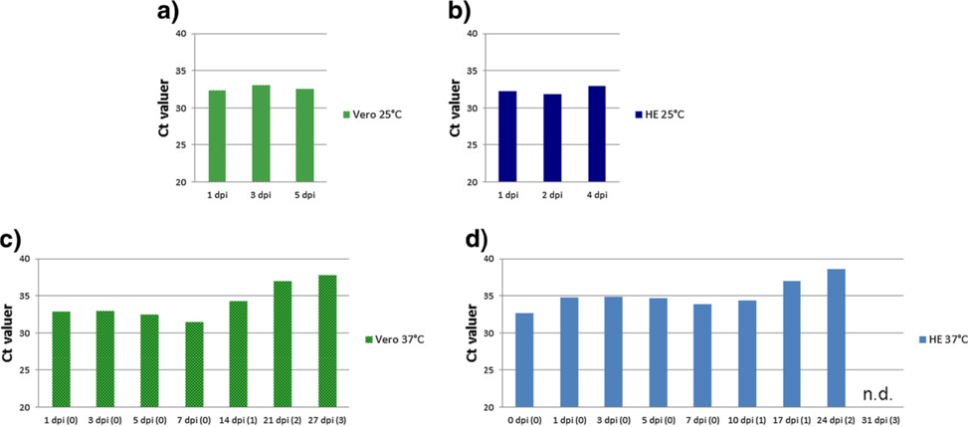
**Detection of IPNV RNA in Vero and HE cells inoculated with IPNV.** Cell culture flasks with Vero cells or HE cells were inoculated with IPNV-1p (TCID_50_ ml^-1^ 10^4^) and incubated in parallels at 25°C (**a** and **b**) and 37°C (**c** and **d**). At the indicated time points, cell monolayers were harvested, nucleic acids extracted, and IPNV RNA detected by RRT-PCR. After one week of incubation, the cells kept at 37°C were passaged onto new flasks and cocultivated (1:1) with noninfected cells. The numbers in parenthesis indicate the passage number. The Ct value is an average of duplicates and n=1. n.d.=not detected.

To investigate whether IPNV replicates in the mammalian cell lines, the amount of viral RNA was followed in infected cell lines over several days and passages. Independent of incubation temperature, the Ct values obtained were relatively stable between 1-5 dpi in the infected Vero cells (Figure 3a) and HE cells (Figure 3b). The Ct values indicate a reduction in the IPNV RNA level between each cell passage of Vero cells (Figure 3c) and HE cells (Figure 3d). Taken together, the results indicate no or only very low levels of IPNV replication in Vero and HE cells. Microscopic examination of the cells prior to the immunolabelling revealed no cpe, and the virus was not detected in supernatant by titration.

## Discussion

The host range of IPNV is very broad, and the virus has been isolated from a wide range of aquatic organisms. The virus also replicates in a variety of piscine cell lines. Other birnaviruses show a more restricted host range both *in vivo* and *in vitro*. IBDV, for instance, is only known to infect poultry and replicates *in vitro* in a few avian primary cell cultures [15], although the virus replicates in several cell lines of mammalian origin [16,17]. The entomobirnavirus DXV most likely also has a narrow host range, as it is thus far only isolated from fruit fly [18]. This indicates that there are important differences in the molecular mechanisms governing the infection processes of these birnaviruses. Investigating the IPNV host range could reveal some of these mechanisms.

Six cell lines of human, leporid, monkey or bovine origin were inoculated with IPNV and investigated by IFAT. Virus specific staining was detected in all cell lines after incubation at 15°C, 25°C and 37°C. IFAT does not distinguish between internalized or surface associated virus. Confocal microscopy analysis was therefore performed on IPNV infected HE and Vero cells. This clearly demonstrated that IPNV was present in the cytoplasm in punctate structures not associated with the plasma membrane. These structures may represent cytoplasmic structures such as endosomes or caveolae [19]. In addition, VOPBA showed that IPNV bound in a specific manner to a membrane protein of approximately 85 kDa from RK-13 cells. Taken together, these findings indicate that IPNV is able to enter into these cells by specific endocytic mechanisms. The difference in molecular size between the IPNV binding proteins from piscine cells and the rabbit cell line could indicate that IPNV attaches to different receptors, and may even represent different entry mechanisms. This, in turn, could explain the broad host range of IPNV.

An early step in viral replication is the production of viral mRNA. IPNV mRNA lacks a poly-A tail, making discrimination between viral genomic RNA and mRNA difficult by PCR. A significant increase in viral RNA over time would, however, indicate that replication has taken place. Thus, the cell cultures were inoculated with low titres of IPNV, and viral RNA levels were detected by RRT-PCR over time. The amount of target nucleic acids was identical in each reaction, and a good correlation was found between the two parallels that were run in the RRT-PCR. Therefore, the Ct values could be used as an indicator of the relative amount of IPNV specific RNA. No significant change in the IPNV RNA level was observed at 5 dpi in HE and Vero cells. As the cells did not grow well at 25°C, incubation at this temperature was terminated at 5 dpi. When the cells were kept at 37°C, on the other hand, they grew to confluence within 7 dpi. No cpe was observed in any of the cell cultures at 7 dpi, even after inoculation with high levels of IPNV. We therefore did not expect any virus in the cell supernatants when inoculating cells with low levels of IPNV. Instead of passaging the virus medium onto fresh cultures, the infected cells cultures were passaged with co-cultivation. For each cell passage, we observed a decrease in the viral RNA levels, most likely due to a dilution of the RNA with each passage. This indicates that no significant level of replication occurs in these cell lines. The gradual decrease in IPNV RNA observed after 2 weeks and longer incubation suggests that the virus multiplication process is arrested before replication of viral nucleic acid starts. Even though the RdRp has been shown to be active at 37°C [8], other factors, such as structural differences between piscine and mammalian proteins or antiviral activity in the mammalian cells, could explain the absence of IPNV replication. The virus may also simply be trapped in the punctate structures described above. When studying RGNNV infection in human cell cultures, Adachi et al. (2008) [10] showed that circumventing some obstacles early in the infection process may lead to the production of progeny RGNNV.

## Conclusion

In conclusion, we have shown that IPNV is able to enter into a wide range of different mammalian cells, and virus entry is most likely receptor mediated. We found no indication of IPNV replication in any of the mammalian cell lines tested.

## Abbreviations

IPNV: Infectious pancreatic necrosis virus; IPN: Infectious pancreatic necrosis; VOPBA: Virus overlay protein binding assay; Dpi: Days post inoculation; IFAT: Indirect immunofluorescent antibody test; Cpe: Cytopathogen effect; IBDV: Infectious bursal disease virus; DXV: Drosophila X virus; BSNV: Blotched snakehead virus; RdRp: RNA dependent RNA polymerase; RGNNV: Red grouper nervous necrosis virus; CHSE: Chinook salmon embryo; RK: Rabbit kidney; FBT: Foetal bovine turbinate; HE: Human embryonic fibroblasts; FBS: Fetal bovine serum; VP: Virus protein; RRT-PCR: Real-time reverse transcription–polymerase chain reaction.

## Competing interests

The authors declare that there are no competing interests.

## Authors’ contributions

IØ conceived the study, contributed to the experimental design, prepared all the virus suspensions and cell membrane protein suspensions, did the cell culture work, VOPBA, RRT-PCR, IFAT, epifluorescence microscopy and image processing, contributed to discussion of the results and drafted the manuscript. TK contributed to the experimental design, did the immunofluorescence, laser confocal microscopy and image processing, and contributed in writing of the manuscript. HS and BD contributed to the experimental design, interpretation of the results and critical reading and revision of the manuscript. ER contributed with discussion of the results and critical reading and revision of the manuscript. All authors read and approved the final manuscript.
